# Supplementary material for: End-to-end protein–ligand complex structure generation with diffusion-based generative models
Source: BMC Bioinformatics. 2023 Jun 5;24:233. doi: 10.1186/s12859-023-05354-5 (PMC10240776; doi:10.1186/s12859-023-05354-5)
Supplement: Supplementary file 1 — Additional file 1. Supplementary information. [file 12859_2023_5354_MOESM1_ESM.pdf]

Supplementary Information for End-to-end protein-ligand complex structure generation with diffusion-based generative model

Shuya Nakata*
shuyanakata@gmail.com

Yoshiharu Mori*
ymori@landscape.kobe-u.ac.jp

Shigenori Tanaka*
tanaka2@kobe-u.ac.jp

The physical accuracy of the generated structures

To validate the physical accuracy of the generated structures, we calculated the distances between the C_α atoms of adjacent residues and compared their distribution with the expected distribution calculated from the experimental structures (Figure S1). All structures generated by DPL on the PDBbind test set (64 structures for each of the 207 complexes) were used for the analysis. Figure S1 shows that the distribution of distances was concentrated around the peak of the expected distribution within a range of ± 0.5 Å. This indicates that the structures generated by DPL are physically plausible, suggesting that a reasonable result could be obtained by building an all-atom model and optimizing its structure using physics-based methods such as Rosetta [1].

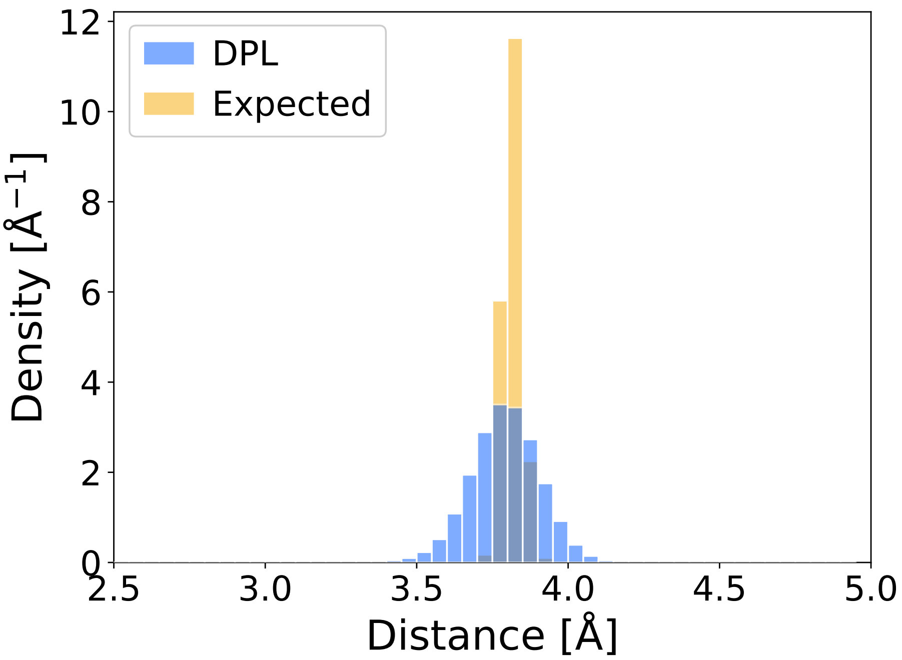

Figure S1: Distribution of distances between C_α atoms of adjacent residues. The distribution calculated from all structures generated by DPL is shown in blue. The expected distribution calculated from the experimental structures is shown in orange.

*Graduate School of System Informatics, Kobe University

Dependence of protein structure reproducibility on training data

Figure S2 illustrates how the amount of training data with similar protein structures (TM-score > 0.5) affects the reproducibility of protein structures. This indicates that the reproducibility increases with the number of related training data, suggesting some overfitting to the protein structures used in the training. However, it also shows that DPL can generalize to proteins with little or no related training data. Extensive use of PDB-registered structures, including apo protein structures, for training would improve the reproducibility of protein structures.

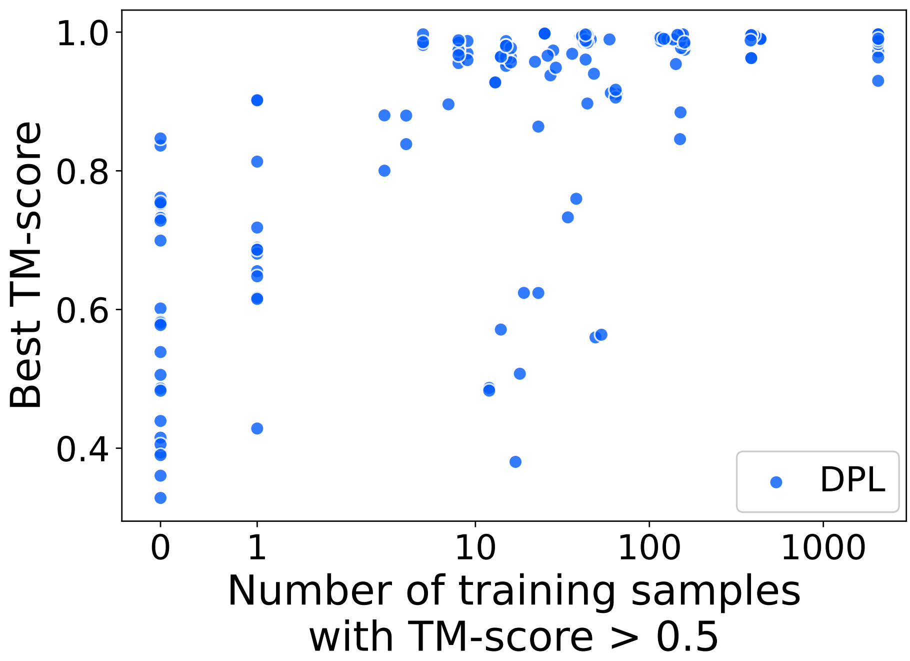

Figure S2: Relation between the protein structure reproducibility represented by TM-score and the number of related training data. Each point represents a complex in the PDBbind test set.

Enhancing training data through random cropping

Randomly cropping partial structures from a large complex is a common method used to enhance the training data in protein structure prediction [2, 3]. For example, the contiguous cropping algorithm described in AlphaFold-Multimer [3] sequentially and randomly crops contiguous residues from each protein chain to fit within a given budget. Although this algorithm is designed for proteins and does not consider the ligand, it can be adapted to protein-ligand complexes by preferentially keeping protein structures in contact with the ligand. An example of such a procedure is shown in Algorithm S1. For this adaptation, contiguous residues are chosen to maximize contact with the ligand, rather than being chosen uniformly at random. One way to define the contact for each chain, $\boldsymbol{c_k} \in \{0,1\}^{n_k}$, is to check if any atom of the residue is within 4 Å of any atom of the ligand. In addition, while the original algorithm randomly shuffles the chains before cropping to avoid bias, it is also possible to reorder them based on contacts with the ligand.

Algorithm S1 Random cropping of a complex structure. Adapted from the contiguous cropping algorithm described in AlphaFold-Multimer [3].

procedure CROP_COMPLEX(Set of chain lengths $\{n_k\}$, Set of chain contacts $\{\boldsymbol{c_k}\}$, The total protein residue budget N_{res})
    Initialize $n_{\text{added}} = 0$
    Initialize $n_{\text{remaining}} = N_{\text{res}}$
    for k in $0, 1, \ldots N_{\text{chains}} - 1$ **do**
        Compute $n_{\text{remaining}} = n_{\text{remaining}} - n_k$
        Compute $\text{crop\_size\_max} = \text{minimum}(N_{\text{res}} - n_{\text{added}}, n_k)$
        Compute $\text{crop\_size\_min} = \text{minimum}(n_k, \text{maximum}(0, N_{\text{res}} - (n_{\text{added}} + n_{\text{remaining}})))$
        Sample $\text{crop\_size} \sim \text{uniform}(\text{crop\_size\_min}, \text{crop\_size\_max} + 1)$
        Compute $n_{\text{added}} = n_{\text{added}} + \text{crop\_size}$
        Compute $\text{crop\_start} = \text{argmax}_s \sum_{i=0}^{\text{crop\_size}} c_{k\,s+i}$
        Initialize $\boldsymbol{m_k} = 0$
        Compute $\text{keep} = [\text{crop\_start}, \ldots, \text{crop\_start} + \text{crop\_size}]$
        Compute $\boldsymbol{m_k}_{\text{keep}} = 1$
    end for
    return $\{m_k\}$
end procedure

References

[1] DiMaio, F., Tyka, M.D., Baker, M.L., Chiu, W., Baker, D.: Refinement of protein structures into low-resolution density maps using rosetta. Journal of Molecular Biology **392**(1), 181–190 (2009). doi:10.1016/j.jmb.2009.07.008

[2] Jumper, J., Evans, R., Pritzel, A., Green, T., Figurnov, M., Ronneberger, O., Tunyasuvunakool, K., Bates, R., Žídek, A., Potapenko, A., Bridgland, A., Meyer, C., Kohl, S.A.A., Ballard, A.J., Cowie, A., Romera-Paredes, B., Nikolov, S., Jain, R., Adler, J., Back, T., Petersen, S., Reiman, D., Clancy, E., Zielinski, M., Steinegger, M., Pacholska, M., Berghammer, T., Bodenstein, S., Silver, D., Vinyals, O., Senior, A.W., Kavukcuoglu, K., Kohli, P., Hassabis, D.: Highly accurate protein structure prediction with AlphaFold. Nature **596**(7873), 583–589 (2021). doi:10.1038/s41586-021-03819-2

[3] Evans, R., O'Neill, M., Pritzel, A., Antropova, N., Senior, A., Green, T., Žídek, A., Bates, R., Blackwell, S., Yim, J., Ronneberger, O., Bodenstein, S., Zielinski, M., Bridgland, A., Potapenko, A., Cowie, A., Tunyasuvunakool, K., Jain, R., Clancy, E., Kohli, P., Jumper, J., Hassabis, D.: Protein complex prediction with AlphaFold-multimer. bioRxiv (2021). doi:10.1101/2021.10.04.463034
